# Large-Kernel Attention for 3D Medical Image Segmentation

**DOI:** 10.1007/s12559-023-10126-7

**Published:** 2023-02-27

**Authors:** Hao Li, Yang Nan, Javier Del Ser, Guang Yang

**Affiliations:** 1 National Heart and Lung Institute, Faculty of Medicine, Imperial College London, London, UK; 2 Department of Bioengineering, Faculty of Engineering, Imperial College London, London, UK; 3 TECNALIA, Basque Research & Technology Alliance (BRTA), Derio, Spain; 4 University of the Basque Country (UPV/EHU), Bilbao, Spain; 5 Royal Brompton Hospital, London, UK

**Keywords:** Attention mechanism, Medical image segmentation, Deep learning

## Abstract

Automated segmentation of multiple organs and tumors from 3D medical images such as magnetic resonance imaging (MRI) and computed tomography (CT) scans using deep learning methods can aid in diagnosing and treating cancer. However, organs often overlap and are complexly connected, characterized by extensive anatomical variation and low contrast. In addition, the diversity of tumor shape, location, and appearance, coupled with the dominance of background voxels, makes accurate 3D medical image segmentation difficult. In this paper, a novel 3D large-kernel (LK) attention module is proposed to address these problems to achieve accurate multi-organ segmentation and tumor segmentation. The advantages of biologically inspired self-attention and convolution are combined in the proposed LK attention module, including local contextual information, long-range dependencies, and channel adaptation. The module also decomposes the LK convolution to optimize the computational cost and can be easily incorporated into CNNs such as U-Net. Comprehensive ablation experiments demonstrated the feasibility of convolutional decomposition and explored the most efficient and effective network design. Among them, the best Mid-type 3D LK attention-based U-Net network was evaluated on CT-ORG and BraTS 2020 datasets, achieving state-of-the-art segmentation performance when compared to avant-garde CNN and Transformer-based methods for medical image segmentation. The performance improvement due to the proposed 3D LK attention module was statistically validated.

## Introduction

Malignant tumors and other organ illnesses have long been a problem for humans, seriously endangering their lives and general well-being. Worldwide, millions of people die from cancer each year, making it the leading cause of mortality [1]. Nevertheless, early identification and therapy are still the most effective means of enhancing cancer survival. Identifying the location of organs and lesions is a crucial step in the diagnostic process and plays a vital role in treating diseases. In general, locating organs and lesions from medical images such as Magnetic Resonance Imaging (MRI) and Computed Tomography (CT) is a segmentation task. Clinicians can determine the location, size, and subtype of a tumor through the precise segmentation of tumors. This benefits not only the diagnostic process but also the planning of radiation therapy or surgery. On the other hand, accurate organ segmentation can help clinicians select personalized treatment strategies for various patients, enabling the practice of precision medicine and individualized care, which can lessen the patient’s financial and psychological burdens. Additionally, the segmentation of longitudinal MRI images can be utilized to track tumor development or shrinkage as well as the response of diseased organs to therapeutic interventions. Therefore, the research and implementation of medical image segmentation are of major significance.

Segmentation of organs and lesions is typically performed manually by experienced radiologists in current clinical practice. Observing medical images to differentiate human organs, tissues, and lesions is a challenging and time-consuming endeavor. Additionally, because manual labeling results rely heavily on the radiologist’s expertise and subjective judgment, they are rarely reproducible and might even involve human bias. Consequently, these problems contribute to the low practicability of manual segmentation. Automated or computer-aided segmentation approaches can solve these issues by requiring less labor and producing objective, reproducible results for later disease diagnosis and management. As a result, automated medical image segmentation has been thoroughly researched and has emerged as the benchmark.

With the increase in computing power and the quick advancement of deep learning technology in recent years, natural image segmentation using fully convolutional neural networks (FCN) [2] has grown rapidly. In the meantime, medical image segmentation remains a formidable challenge, as medical images are characterized by uneven grayscale, significant contrast variation, and substantial noise. Since U-Net [3] was published, medical image semantic segmentation has also undergone tremendous development.

However, the existing technology for the automated segmentation of medical images lacks sufficient intelligence and precision. For multi-organ segmentation, it is inherently challenging to differentiate between different organs due to overlapping boundaries and intricate connections. Moreover, the wide variation in anatomy and low contrast between tissues makes the segmentation task more ambiguous and complex to tackle [4–6]. For lesion segmentation, although the tumor does not have any problems in terms of overlapping, lesions can arise in any position, unlike organs, which are relatively fixed spatially. In addition, tumors exhibit a wider range of sizes, shapes, and appearances [7]. Furthermore, in many cases, the tumor volume is rather small relative to the entire scan, resulting in the dominance of the background noise [8]. All of these issues lower the segmentation accuracy. In clinical practice, even minute inaccuracies in medical image segmentation might result in misdiagnosis. Therefore, segmentation models based on deep learning have significant room for development in this discipline.

Long-range self-attention can be used to enable the network to learn only the truly crucial information [9], such as the organ boundaries or tumor-related features. It is an attention mechanism for adaptive input selection based on the inputs’ features. In deep learning, attention is a biologically inspired technique designed to mimic cognitive attention [10]. Different self-attention techniques have been used in medical image segmentation [11–13]. They have obtained superior performance compared to traditional FCNs because of their efficiency in capturing long-range dependencies. Despite recent attempts [11–13], self-attention has several shortcomings when it comes to medical image segmentation since it was designed for Natural Language Processing (NLP). First, it analyzes images as one-dimensional sequences, ignoring the structural details required for obtaining morphological features in medical images. Second, since 3D scans like MRI or CT are too computationally expensive with quadratic complexity, most self-attention research is 2D-based. Third, it disregards the necessity of channel adaptation for attention processes. For semantic segmentation tasks, different channels usually represent features of different objects. Thus, adaptation in channel maps is important for attention to build dependencies within channels [12, 14, 15].

In order to address these issues, this paper introduces a novel large-kernel (LK) attention module for enhancing medical image segmentation. The LK attention module combines self-attention and convolution’s advantages, such as long-range dependencies, spatial adaptation, and local contextual information, and avoids their disadvantages, such as disregarding channel adaptation and computational complexity. In this way, we can spatially focus on details related to the segmentation target, such as organs or tumors, in a holistic view. Moreover, since each channel usually represents the features of each organ (as shown in Fig. 5), the spatial and channel adaptability makes the feature extraction more precise and specific to each organ, which helps resolve the ambiguity caused by overlapping boundaries between organs. This paper is based on our previous work on MRI brain tumor segmentation at the Medical Image Understanding and Analysis Conference (MIUA) [16]. On this basis, we optimized the LK attention model, conducted comprehensive ablation experiments to demonstrate its feasibility, and explored more efficient design and deployment strategies. We also further investigated whether LK attention could improve the performance of CT multi-organ segmentation to expand the application scope and adaptability of LK attention in medical imaging and segmentation tasks. The following highlights the key contributions of this paper:

- A novel 3D LK attention utilizing decomposed LK convolutions was proposed, which combines the advantages of convolution and self-attention while avoiding their disadvantages.
- A U-Net architecture that efficiently incorporates 3D LK attention was proposed for the segmentation of 3D medical images. By adaptively amplifying the weights of key features while reducing the weights of noisy voxels and channels, the 3D LK attention-based U-Net can accurately identify the location of various organs and tumor subregions.
- In publicly available datasets for evaluating multi-organ and tumor segmentation, 3D LK attention-based U-Net outperformed state-of-the-art methods in delineating all targets.
- Extensive ablation experiments were performed, and the findings validated the effectiveness of the decomposition of the 3D LK convolution and investigated the optimal deployment and design strategies for the 3D LK attention module.
- The proposed 3D LK attention module is easy to integrate into any other neural network. Quantitative studies demonstrated that it could effectively improve the accuracy of 3D medical image segmentation and provide local explanations.

The rest of the article is structured as follows: “Related Work” section will briefly review related work. “Method” section will detail our segmentation method, including the LK attention module and network architecture. “Experiment” section will illustrate the experimental setup, and results and discussion will be presented in “Results and Discussion” section. The conclusion will be given in the final “Conclusion” section.

## Related Work

In this section, we will briefly review the recent work related to multi-organ segmentation (“Multi-organ Segmentation” section) and tumor segmentation (“Tumor Segmentation” section), including some applications of self-attention. We will also review recent work that adopted the large kernels and comparatively present our contribution (“Large Kernels” section).

### Multi-organ Segmentation

Multi-organ segmentation, which comprehensively classifies voxels into multiple organ classes rather than just organs or other tissues, gives a broader viewpoint on the task of organ segmentation. This involves identifying which organ type a particular voxel belongs to, in addition to determining if it belongs to an organ. Due to the increased data volume and image complexity, the automated segmentation of multiple organs in 3D medical images is challenging.

A method for segmenting 3D CT images using majority voting was proposed in [17] based on the FCN. In [18], a neural network dubbed 3D DSN avoids unnecessary computation and overfitting via volume-to-volume learning, making it suited for applying to cardiac and hepatic anatomy. Roth et al. [19] presented a coarse-to-fine method for multi-organ segmentation that included two stages. The 3D FCN in the first stage extracts candidate regions coarsely, whereas the second 3D FCN focuses on potential organ region boundaries in a cascaded way, hence minimizing the number of voxels to be processed. Similar research was conducted by [20] employing cascaded 3D FCNs for dual-energy CT. [21] presented a 3D-U-JAPA-net based on transfer learning, whereas [22] created a semi-supervised network to fully exploit the unlabeled data. To save GPU memory, [23] suggested combining 2D and 3D models, performing segmentation using 2D convolutions and extracting spatial information from 3D models.

To comprehensively benchmark multi-organ segmentation methods for the abdomen, the first Fast and Low GPU Memory Abdominal Organ Segmentation (FLARE) challenge was recently organized [24]. In this challenge, 23 methods are benchmarked on a large and diverse dataset of abdominal CT, including 511 cases from 11 medical centers. The winning method outperforms the baseline with 19 times faster inference, using coarse-to-fine U-Nets with mixed pyramid pooling [25]. Although FCNs have been proven to be very successful, learning long-range spatial relationships is challenging due to the localization of convolutional layers. The UNETR architecture was proposed by [26], who was inspired by transformers used in NLP. The transformer acting as an encoder enables U-Net to collect global information and model long-range spatial relationships, leading to superior segmentation results. However, it converted the 3D anatomical structure segmentation to a sequence-to-sequence prediction problem.

### Tumor Segmentation

Identification of tumors can be aided by image analysis across various imaging modalities. The Brain Tumor Segmentation Challenge (BraTS) compiles a well-known public multi-modal MRI dataset. The BraTS challenge compares cutting-edge brain tumor segmentation methods annually [27–29]. T1-weighted (T1), post-contrast T1-weighted (T1ce), T2-weighted (T2), and T2 fluid attenuated inversion recovery (FLAIR) 3D MRI modalities are available for each patient case.

Since 2014, deep learning algorithms have been extensively researched for tumor segmentation in the BraTS challenge [9, 30–39]. Myronenko [32] won the BraTS 2018 competition by training an asymmetrical U-Net with a broader encoder and an additional variational decoder branch that provided further regularization. A two-stage cascaded asymmetrical U-Net comparable to Myronenko [32] was proposed by Jiang et al. [34]. The first step generated a coarse prediction, whereas the second stage utilized a larger network to refine the outcome. In order to automatically adapt the traditional U-Net to a particular dataset with just minor alterations, Isensee et al. [33] adopted a self-configuring framework called nnU-Net. Wang et al. [35] suggested a modality-pairing learning method that uses the layer connection on parallel branches to extract the complicated interactions and rich information between various MRI modalities.

A recent study [37] proposed an optimized U-Net architecture for the BraTS challenge. To find the optimal architecture and learning strategy, extensive ablation studies were conducted to test: U-Net depth, number of convolutional channels, decoder attention, residual connections, losses, and post-processing strategy. Similarly, [38] developed a novel brain tumor segmentation method by improving nnU-Net, including using a larger network, replacing batch normalization with group normalization, and using axial attention in the decoder. In addition, [39] proposed a trusted brain tumor segmentation network, which could generate robust segmentation results and reliable uncertainty estimates, modeled using subjective logic theory. The trusted framework learns to gather evidence from the features, endowing the model with reliability for out-of-distribution samples. Jia et al. [9] created the Hybrid High-resolution and Non-local Feature Network (H2NF-Net), which used parallel multi-scale convolutional blocks to utilize multi-scale features and preserve high-resolution features representation simultaneously. The self-attention mechanism implemented in this study permits the aggregation of local information across spatial locations and the acquisition of long-range dependencies. However, this attention can only operate on a set of feature reconstruction bases rather than high-resolution feature maps.

### Large Kernels

In the recent past, the contribution of large kernels to natural image segmentation was first highlighted in [40]. However, instead of using additional large kernels to capture long-range dependencies, this paper directly used large kernels to extract features, so a refinement module was required. This idea was extended by [41], using re-parameterization to scale up the kernels to $31 \times 31$. The proposed RepLKNet [41] achieved comparable or better results than transformers on classification, semantic segmentation, and object detection of natural images. On the other hand, Yang et al. [42] utilized large kernels to improve the performance of spatial pyramid pooling and demonstrated the improvement in the road extraction task.

Several concurrent works also adopted large kernels as attention mechanisms, including LKASR [43] for lightweight image super-resolution and LKD-Net [44] for single Image dehazing. However, all related papers only proved that large kernels were effective on natural images, and no study had attempted to employ large kernels in 3D due to computational cost constraints as mentioned in the previous section. The only attempt at medical image segmentation was [45], which proposed an anisotropic network for MRI brain tumor segmentation. This paper ingeniously combined 2D large-kernel convolutions on two different axes to achieve anisotropic 3D segmentation, but also ignored the holistic 3D anatomical structure.

Therefore, this work is the first to demonstrate the feasibility and effectiveness of 3D large-kernel attention for a variety of segmentation tasks across different medical image modalities. We present guidelines on how to efficiently implement 3D large-kernel attention and show that it is able to provide 3D local explanations that are only reasonable for three-dimensional medical scans.

## Method

Our method is detailed in this section, including the new LK attention module (“LK Attention” section) and the modified U-Net based on the LK attention module for 3D medical image segmentation (“LK Attention-Based U-Net” section).

### LK Attention

Numerous studies have demonstrated that the integration of diverse attention mechanisms has the potential to enhance segmentation performance. The attention map reflects the relative significance across the feature space, which necessarily involves the capture of correlations between various locations. The self-attention can be used to discover long-range dependencies, but it has several disadvantages, as stated in the previous section. Applying large-kernel convolution to establish long-distance dependencies and generate the attention map is an alternative method [14, 15, 46–49]. Nevertheless, this strategy substantially increases the computational cost.

**Fig. 1:**
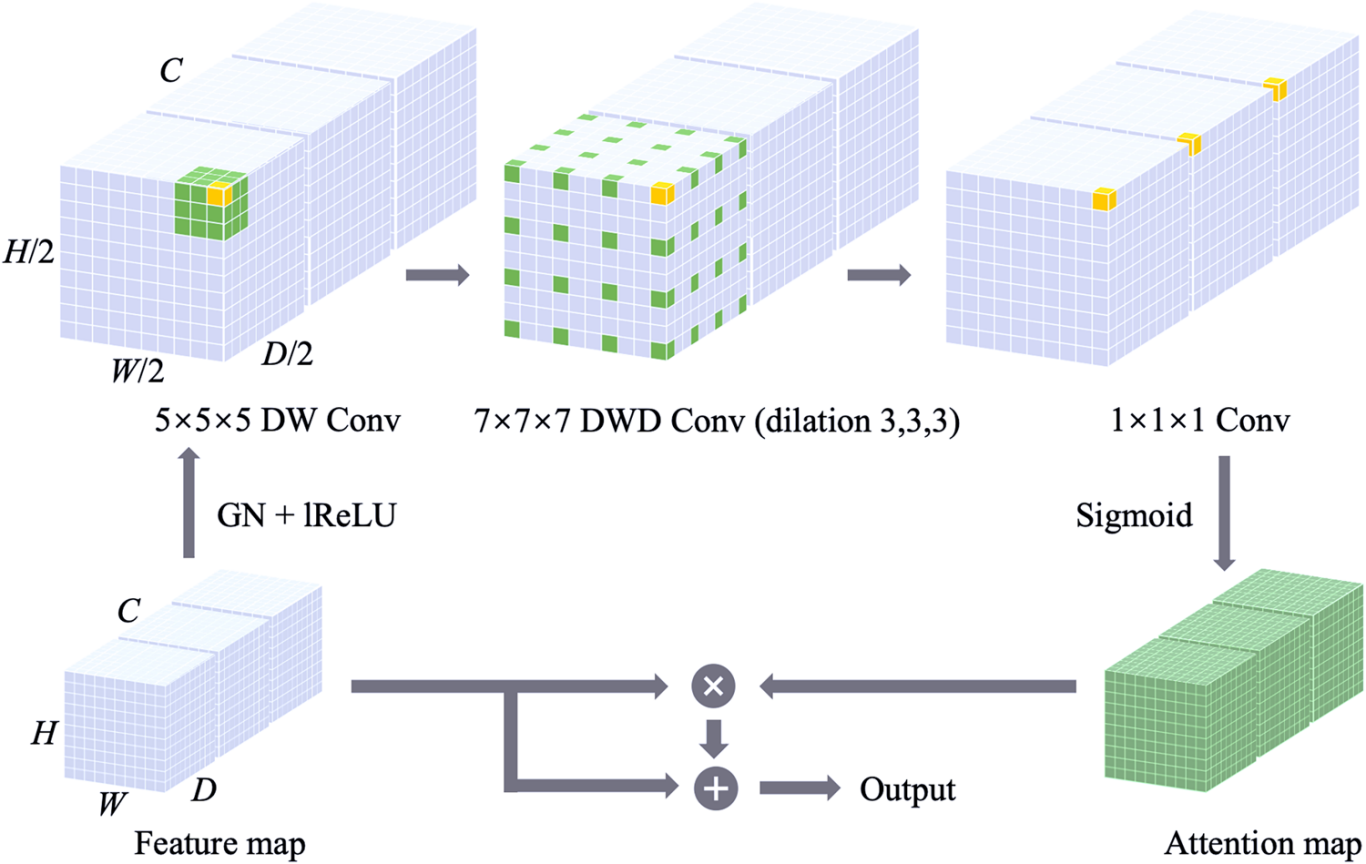
LK attention module. The decomposed LK convolution is applied on the feature map after group normalization (GN) and leaky ReLU (lReLU). The attention map is obtained by sigmoid activation, which is then multiplied and summed elementwise with the original feature map to generate the module output. The figure shows a representative decomposition of a $21\times 21\times 21$ convolution into a $5\times 5\times 5$ depth-wise (DW) convolution, a $7\times 7\times 7$ depth-wise dilated (DWD) convolution with dilation of 3, and a $1\times 1\times 1$ convolution. The position of the kernel is indicated by colored voxels, and the yellow voxels show the kernel’s centers. (The figure only illustrates a corner of the feature space of the decomposed LK convolution and disregards the zero-padding)

To address these limitations and maximize the benefits of self-attention and large-kernel (LK) convolution, we developed an LK attention module (shown in Fig. 1). Assuming *K* is the number of channels, a $K\times {K} \times {K}$ LK convolution was decomposed into a $(2d-1)\times (2d-1)\times (2d-1)$ depth-wise (DW) convolution, a $\frac{K}{d}\times \frac{K}{d}\times \frac{K}{d}$ depth-wise dilated (DWD Conv) convolution with dilation of d and a $1\times 1\times 1$ convolution. For an input with dimensions of $H\times W\times D\times C$, the number of parameters (${\text {N}}_{\text {PRM}}$) and the number of floating-point operations (FLOPs) for the original LK convolution and its decomposition can be calculated as follows:

1 $$\begin{aligned} {\text {N}}_{\text {PRM,O}}=C\times (C\times (K\times K\times K)+1), \end{aligned}$$

2 $$\begin{aligned} {\text {FLOPs}}_\text {O}=C\times (C\times (K\times K\times K)+1)\times H\times W\times D, \end{aligned}$$

3 $$\begin{aligned} \begin{aligned} {\text {N}}_{\text {PRM,D}}=&\;C\times ((2d-1)\times (2d-1)\times (2d-1)\\&+\frac{K}{d}\times \frac{K}{d}\times \frac{K}{d}+C+3), \end{aligned} \end{aligned}$$

4 $$\begin{aligned} \begin{aligned} {\text {FLOPs}}_\text {D}=&\;C\times ((2d-1)\times (2d-1)\times (2d-1)\\&+\frac{K}{d}\times \frac{K}{d}\times \frac{K}{d}+C+3)\times H\times W\times D, \end{aligned} \end{aligned}$$

where O and D represent the original LK convolution and decomposed LK convolution, respectively. To determine the optimal d such that $N_{PRM}$ is minimal for a particular kernel size K, we set the first derivative of Eq. (3) to 0 and then solved as follows:

5 $$\begin{aligned} \frac{d}{dd^*}\left( C\left( \left( 2d^*-1\right) ^3+\left( \frac{K}{d^*}\right) ^3+C+3\right) \right) =0, \end{aligned}$$

6 $$\begin{aligned} 24d^2-24d-\frac{3K^3}{d^4}+6=0. \end{aligned}$$

In Eq. (5), the superscript $*$ distinguishes dilation *d* from derivation *d*. For $K=21$, solving Eq. (5) numerically yielded an optimal approximation of *d* of approximately 3.4159. As shown in Table 1, the number of parameters can be significantly lowered with a dilation rate of 3. We can also observe that as the number of channels increases, the decomposition becomes more efficient.

**Table 1:** Complexity analysis: comparison of the number of parameters $N_{PRM}$ for a $21\times 21\times 21$ convolution

*C*	${\text {N}}_{\text {PRM,O}}$	${\text {N}}_{\text {PRM,D}}$	${\text {N}}_{\text {PRM,D}}/{\text {N}}_{\text {PRM,O}}$
32	9.48 M	16.10 k	0.17%
64	37.94 M	34.24 k	0.09%
128	151.75 M	76.67 k	0.05%
256	606.99 M	186.11 k	0.03%
512	2427.98 M	503.30 k	0.02%

The subscripts *O* and *D* denote the original convolution and the proposed decomposed convolution, respectively. *C*: number of channels

The entire LK attention module is formulated as follows:

7 $$\begin{aligned} A=\sigma _{\text {sigmoid}}\left( {\text {Conv}}_{1\times 1\times 1}\left( {\text {Conv}}_{\text {DW}}\left( {\text {Conv}}_{\text {DWD}}\left( \sigma _{\text {lReLU}}\left( \text {GN}\left( Input\right) \right) \right) \right) \right) \right) , \end{aligned}$$

8 $$\begin{aligned} Output=A\otimes \left( \sigma _{\text {lReLU}}\left( \text {GN}\left( Input\right) \right) \right) +\sigma _{\text {lReLU}}\left( \text {GN}\left( Input\right) \right) , \end{aligned}$$

where *A* denotes the attention map, and GN is the group normalization. $\sigma _{\text {lReLU}}$ and $\sigma _{\text {sigmoid}}$ denote to leaky ReLU activation function and sigmoid activation function, respectively. The LK Attention module’s output is formed by multiplying and summing the input feature map and the attention map element by element. Using the LK attention module, we can extract long-range dependencies within a feature space and generate the attention map with minimal computing complexity and parameters.

### LK Attention-Based U-Net

**Fig. 2:**
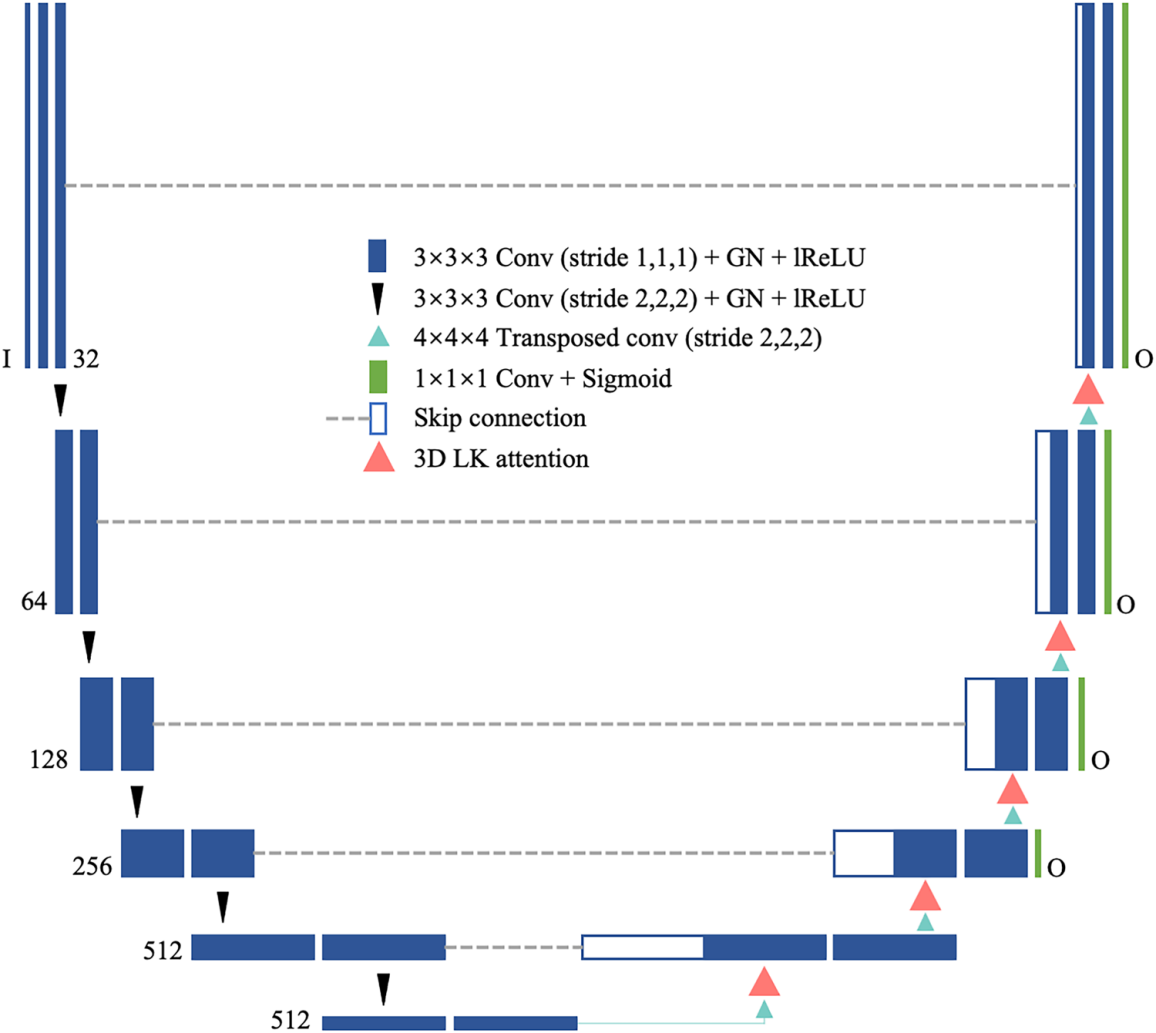
The network architecture of our proposed LK attention-based U-Net

The U-Net [3] has served as a basis for numerous studies on medical image processing. Its capacity to capture fine object features utilizing skip connection is particularly advantageous for precise segmentation. As shown in Fig. 2, the 3D LK attention-based U-Net architecture is based on the U-Net and comprises an encoding path of feature extraction and a decoding path of inference with the skip connection.

#### Encoder

The encoder is composed of convolution blocks of six scales. Each block contains two convolution layers with a $3\times 3\times 3$ kernel, GN, and lReLU (with a slope of 0.01). The input data of *I* channels is convoluted by 32 kernels to generate the initial 32 feature maps, and the channel number *I* corresponds to the number of imaging modalities. Between the two scales, a stride-2 $3\times 3\times 3$ convolution is used to downsample the feature map by 2 and increase the number of channels to a maximum of 512. The deepest feature map is 1/32 of the original size.

#### LK Attention-Based Decoder

The architecture of the decoder is identical to that of the encoder, using $4\times 4\times 4$ transposed convolution for upsampling. The LK attention module can be applied to each upsampled feature map to form a fully applied (Full) network as in our previous paper. The details of the LK attention module for the Full network are shown in Table 2. At the last layer, a $1\times 1\times 1$ convolution is applied to compress the channel number O according to the number of segmentation classes, followed by the softmax/sigmoid to generate probability maps for different organs or tumor regions. Additional softmax/sigmoid outputs were added to all scales except the two lowest levels for deep supervision and boost gradient propagation.

**Table 2:** Details of LK attention modules in the Full LK attention-based U-Net

**Scale**	**DW Conv**		**DWD Conv**			**Equal LK Conv**
	Kernel	Padding	Kernel	Dilation	Padding	Kernel
10$\times$12$\times$8	(3, 3, 3)	(1, 1, 1)	(3, 3, 3)	(2, 2, 2)	(2, 2, 2)	(6, 6, 6)
20$\times$24$\times$16	(3, 3, 3)	(1, 1, 1)	(3, 3, 3)	(2, 2, 2)	(2, 2, 2)	(6, 6, 6)
40$\times$48$\times$32	(3, 3, 3)	(1, 1, 1)	(5, 5, 5)	(2, 2, 2)	(4, 4, 4)	(10, 10, 10)
80$\times$96$\times$64	(5, 5, 5)	(2, 2, 2)	(5, 5, 5)	(3, 3, 3)	(6, 6, 6)	(15, 15, 15)
160$\times$192$\times$128	(5, 5, 5)	(2, 2, 2)	(7, 7, 7)	(3, 3, 3)	(9, 9, 9)	(21, 21, 21)

## Experiment

The LK attention is evaluated on standard benchmarks: CT-ORG [50] for multi-organ segmentation and BraTS 2020 for tumor segmentation. We first conducted extensive ablation experiments to evaluate the proposed module’s effectiveness thoroughly.

### Data Acquisition

The CT-ORG [50] dataset consists of 140 CT images of six organ classes, including liver, lungs, bladder, kidneys, bones, and brain. Of the total 140 image volumes, 131 were dedicated CTs, and 9 were CT components collected during PET-CT examinations. Each image was acquired from a different patient. Most images displayed benign or malignant liver lesions; some showed metastasis from breast, colon, bone, and lung cancers. The images were collected from a variety of sources, including low-dose, high-dose, contrast, and non-contrast CT, with dedicated CTs ranging from 0.56 to 1 mm in axial resolution. Some images were received from the Liver Tumor Segmentation Challenge (LiTS) [51]. With the help of ITK-SNAP and morphological segmentation, manual labeling of soft tissues was conducted for all images. However, the manual correction for annotations of lungs and bones was only conducted on the test dataset (comprising 21 cases). Therefore, we tested our model on these 21 cases as suggested by the provider of the dataset.

The BraTS 2020 dataset was collected using various clinical protocols and scanners from different institutions. The ground truth (GT) labels are annotated by one to four raters and approved by specialists, which include the GD-enhancing tumor (ET), peritumoral edema (ED), and necrotic and non-enhancing tumor core (NCR + NET). The segmentation results are evaluated on three subregions of the tumor: the GD-enhancing tumor (ET), the tumor core (TC = ET + NCR + NET), and the whole tumor (WT = ET + NCR + NET + ED). The image modalities T1, T1ce, T2, and T2-FLAIR are co-registered to the same template with an image size of $240\times 240\times 155$. Afterward, they are interpolated to the same resolution ($1\,\text {mm}^3$) and skull-stripped. Annotations are only available for the training set (369 cases). The evaluation of the independent validation set (125 cases) should be conducted on the official online platform (CBICA’s IPP^1^). Details of the two datasets are summarized in Table 3.

**Table 3:** Details of datasets

**Dataset**	**Modality**	**Labels**	**Classes**	**Training set**	**Test set**
CT-ORG	CT	Organs	6	119	21
BraTS 2020	MRI (4 modalities)	Brain tumors	3	369	125

### Pre-processing and Data Augmentation

For the CT-ORG dataset, our network takes an image volume of $128\times 128\times 256$ as input. To reduce GPU memory usage, all image volumes were resampled to $3~\text {mm}^3$. Resampling uses Gaussian smoothing to avoid aliasing artifacts, followed by resolution interpolation. All image volumes for the BraTS 2020 dataset are cropped to $160\times 192\times 128$ to reduce computational waste on background voxels. All input volumes are then pre-processed by intensity normalization.

Various data augmentation techniques have been applied to artificially increase dataset size and minimize the risk of overfitting. All augmentations are applied on-the-fly throughout the training to expand the training dataset indefinitely. Furthermore, to increase the variability of the generated data, all augmentations are applied randomly based on preset probabilities, and most parameters are also drawn randomly (see Table 4 for details).

**Table 4:** Details of data augmentation strategies

**Methods**	**Probability**	**Range**
Brightness	30%	*U*(0.7, 1.3)
Contrast	15%	*U*(0.6, 1.4)
Gaussian Noise	15%	variance $\sigma \sim U (0, 1)$
Gaussian Blur	20%	kernel $\sigma \sim U (0.5, 1.5)$
Gamma Augmentation	15%	$\gamma \sim U(0.7, 1.5)$
Scaling	30%	*U*(0.65, 1.6)
Rotation	30%	$U(-30, 30)$
Elastic Transform	30%	$\alpha \sim U(5, 10), \sigma =3\alpha$
Flipping	50%	along all axes

### Training and Optimization

The LK attention-based U-Net is trained separately on CT-ORG and BraTS 2020 training datasets. For the CT-ORG training set (119 cases), the network parameters are optimized for weighted soft Dice loss. The weight for each segmentation class is one minus the ratio of foreground voxels to background voxels. For the BraTS 2020 training set (369 cases), binary cross-entropy (BCE) and soft Dice losses are utilized.

The adaptive moment estimator (Adam) optimizer was applied to optimize the parameters of the network. Each training process had 200 epochs with a batch size of 1 and an initial learning rate of 0.0003. All experiments were implemented with Pytorch 1.10 on an NVIDIA GeForce RTX 3090 GPU of 24GB VRAM.

### Evaluation Metrics

The segmentation results were evaluated using the Dice score and 95 percent Hausdorff distance (HD95), which are defined as:

9 $$\begin{aligned} \text {Dice} = \frac{2|\mathcal {X} \cap \mathcal {Y} |}{|\mathcal {X} |+|\mathcal {Y}|}, \end{aligned}$$

10 $$\begin{aligned} \text {HD95} = P_{95}\left( \max \left( \max _{x\in \mathcal {X} }{\min _{y\in \mathcal {Y}}{|y-x|}},\max _{y\in \mathcal {Y}}{\min _{x\in \mathcal {X} }{|x-y|}}\right) \right) , \end{aligned}$$

where $\mathcal {X}$ and $\mathcal {Y}$ are sets of GT and prediction, and P represents the percentile. HD95 indicates the 95th percentile of maximum distances between two boundaries, whereas the Dice score measures spatial overlap between the segmentation result and the GT annotation. The final performance of LK attention-based U-Net was evaluated using independent test sets from CT-ORG (21 cases) and BraTS 2020 (125 cases), respectively. The brain class was excluded from evaluation because only 8 of the 119 training CT images had complete coverage of the patient’s head.

## Results and Discussion

This section will first experimentally demonstrate the effectiveness of our LK attention module design (“Qualitative Analysis of Ablation Experiments” section), and then quantitatively analyze the segmentation results (“Quantitative Analysis of Segmentation” section). The limitations of the proposed method will be also discussed in the last subsection (“Limitations” section).

### Qualitative Analysis of Ablation Experiments

For the ablation study, the CT-ORG test dataset was used for evaluation, and the network without any attention module was adopted as the base model. We first verify the effectiveness of LK convolutional decomposition and then look for efficient ways to compute the attention map through different model variants.

We conducted ablation experiments by adding different single attention modules to the base network. By comparing the attention module using the original LK convolution with the attention module using the decomposed LK convolution, the decomposition of the LK convolution was proven to be effective and efficient. The comparative results in Table 5 show that the segmentation results of the two attention modules were very close at both the deepest and shallowest levels. The changes in the averaged Dice score were not significant, verified by paired t-tests in the test set, giving p-values of 0.094 and 0.122, respectively. On the other hand, we can see that the decomposition of LK convolution significantly reduced the number of added parameters to about 0.5% and 0.2% of the original, respectively.

**Table 5:** Quantitative results to compare the decomposed (D) 3D LK convolution with the original (O) 3D LK convolution

**Scale**	**LK Conv**	${\textbf {N}}_{{\textbf {PRM}}}$**(k)**	**Dice**$\uparrow$
			mean
None (Base)	N/A	101017.22	91.43 (1.66)
10$\times$12$\times$8	O	+56623.62	90.72 (2.30)
10$\times$12$\times$8	D	+291.33	90.63 (2.07)
160$\times$192$\times$128	O	+9483.30	91.20 (1.98)
160$\times$192$\times$128	D	+16.10	91.25 (1.95)

Metrics are shown as mean (standard deviation). mean: averaged Dice scores of all organs/subregions

The LK attention module can be applied to each upsampled feature map. However, the additional computational cost of a fully applied (Full) network is high, and the efficiency of its design deserves to be analyzed. Therefore, we explored many variants of attention modules with different sizes and positions, as shown in Table 6. Applying decomposed LK attention modules with different kernel sizes at the same location ($160\times 192\times 128$) indicated that larger kernel coverage leads to better segmentation performance. Kernel coverage refers to the ratio of the kernel size to the feature space size. This is reasonable because convolutions with larger kernels capture correlations across longer distances more effectively. While decomposed LK convolutions with the same kernel size (6, 6, 6) at different locations show that the LK attention module worked best in the middle of the decoder. We can see that when the LK attention module of fixed kernel size was applied to larger scales, its segmentation performance initially increased but then started to decrease slightly due to the significant reduction of kernel coverage at high levels. Therefore, to balance the effects of kernel size and position, we applied the largest LK attention module in the middle, which achieved the highest Dice score. This observation was statistically verified by pair t-tests as shown in Table 6. To conclude, the network structure utilizing LK attention in the middle of the decoder (Mid) is the most effective and efficient, with the number of added parameters being nearly one-sixth of the Full network.

**Table 6:** Quantitative results to compare 3D LK attention modules of different kernel sizes at different locations in the network

**Scale**	**Equal LK Conv**	**Kernel Coverage**	**NPRM (k)**	**Dice**$\uparrow$
				mean
None (Base)	N/A	N/A	101017.22	91.43 (1.66)^a^
10$\times$12$\times$8	(6, 6, 6)	22.50%	+291.33	90.63 (2.07)^a,b^
20$\times$24$\times$16	(6, 6, 6)	2.81%	+80.13	90.84 (1.90)^a,b^
40$\times$48$\times$32	(6, 6, 6)	0.35%	+23.68	91.32 (2.24)^a^
80$\times$96$\times$64	(6, 6, 6)	0.04%	+7.74	91.02 (1.13)^a^
160$\times$192$\times$128	(6, 6, 6)	0.01%	+2.85	90.61 (2.32)^a,b^
160$\times$192$\times$128	(10, 10, 10)	0.03%	+5.98	90.83 (2.28)^a,b^
160$\times$192$\times$128	(15, 15, 15)	0.09%	+9.12	91.13 (1.85)^a,b^
160$\times$192$\times$128	(21, 21, 21)	0.24%	+16.10	91.25 (1.69)^a^
40$\times$48$\times$32 (Mid)	(21, 21, 21)	15.07%	+76.67	92.15 (1.50)^b^
All (Full)	see Table 2	N/A	+444.06	91.69 (1.81)^a^

Metrics are shown as mean (standard deviation). mean: averaged Dice scores of all organs/subregions

^a^p-value < 0.05 compared with Mid network by paired t-test

^b^p-value < 0.05 compared with Base network by paired t-test

### Quantitative Analysis of Segmentation

The evaluation of the segmentation performance of the proposed methods was conducted and compared with state-of-the-art methods, including CBAM [15] using an independent CT-ORG test set (21 cases) and BraTS 2020 validation set (125 cases), which are shown in Tables 7, 8, 9, and 10.

**Table 7:** Quantitative results (Dice) of proposed methods compared to state-of-the-art methods for CT-ORG

**Method**	**Dice**$\uparrow$					
	liver	bladder	lungs	kidneys	bone	mean
U-Net [3]	94.83 (2.56)^a^	76.79 (17.89)^a^	93.85 (4.55)^a^	89.35 (4.01)^a^	85.43 (6.27)^a^	88.05 (5.77)^a^
nnU-Net [33]	95.48 (1.45)^a^	85.00 (3.69)^a^	95.21 (3.47)^a^	91.74 (2.86)^a^	87.84 (3.15)^a^	91.25 (1.89)^a^
UNETR [26]	95.88 (1.24)^a^	86.20 (3.20)^a^	96.21 (2.66)^a^	91.98 (1.34)^a^	88.01 (1.71)^a^	91.33 (1.72)^a^
Ours (Base)	95.81 (1.63)^a^	**86.81 (2.91)**	94.23 (2.86)^a^	92.11 (2.13)^a^	88.20 (2.00)^a^	91.43 (1.20)^a^
Ours (CBAM)	95.92 (1.27)^a^	86.63 (3.58)	94.48 (2.94)^a^	91.57 (1.94)^a^	88.15 (1.93)^a^	91.35 (1.69)^a^
Ours (Full)	96.12 (1.10)	86.63 (2.93)	95.56 (2.00)^a^	91.70 (2.08)^a^	88.45 (1.31)	91.69 (1.81)^a^
**Ours (Mid)**	**96.12 (1.07)**	86.48 (2.68)	**97.40 (1.85)**	**92.26 (1.46)**	**88.51 (1.99)**	**92.15 (1.50)**

Metrics are shown as mean (standard deviation). mean: averaged Dice scores of all organs/subregions. Bold numbers are the best results

^a^p-value < 0.05 compared with Mid network by paired t-test

**Table 8:** Quantitative results (HD95) of proposed methods compared to state-of-the-art methods for CT-ORG

**Method**	**HD95**$\downarrow$					
	liver	bladder	lungs	kidneys	bone	mean
U-Net [3]	3.71 (4.56)^a^	4.64 (8.33)^a^	14.10 (9.49)^a^	4.87 (2.92)^a^	6.27 (2.53)^a^	6.52 (6.07)^a^
nnU-Net [33]	1.81 (2.69)^a^	3.02 (2.55)^a^	9.67 (5.82)^a^	3.11 (1.94)^a^	4.25 (1.30)^a^	4.35 (3.67)^a^
UNETR [26]	1.60 (1.79)^a^	3.05 (3.03)^a^	8.93 (6.38)^a^	3.44 (1.84)^a^	4.77 (1.61)^a^	4.18 (2.90)^a^
Ours (Base)	1.64 (1.54)^a^	**2.83 (3.04)**	10.38 (5.63)^a^	2.90 (1.65)^a^	4.93 (1.72)^a^	4.52 (2.68)^a^
Ours (CBAM)	1.55 (1.57)	2.99 (3.39)	10.00 (5.46)^a^	3.68 (2.89)^a^	4.43 (1.54)^a^	4.53 (2.37)^a^
Ours (Full)	1.56 (1.75)	2.97 (3.00)	9.56 (5.26)^a^	3.24 (2.44)^a^	4.40 (1.47)^a^	4.35 (2.65)^a^
**Ours (Mid)**	**1.53 (1.56)**	2.93 (2.85)	**6.54 (5.30)**	**2.80 (1.81)**	**4.12 (1.43)**	**3.64 (2.23)**

Metrics are shown as mean (standard deviation). mean: averaged Dice scores of all organs/subregions. Bold numbers are the best results

^a^p-value < 0.05 compared with Mid network by paired t-test

**Table 9:** Quantitative results (Dice) of proposed methods compared to state-of-the-art methods for BraTS 2020

**Method**	**Dice**$\uparrow$			
	ET	WT	TC	mean
U-Net [3]	64.77 (31.80)^a^	84.31 (8.98)^a^	72.61 (23.00)^a^	73.90 (16.31)^a^
nnU-Net [33]	77.07 (12.80)^a^	90.10 (2.52)^a^	84.26 (3.89)^a^	83.81 (3.99)^a^
UNETR [26]	78.15 (13.06)^a^	90.29 (2.04)^a^	84.46 (4.25)^a^	84.30 (3.58)^a^
Ours (Base)	77.94 (11.67)^a^	90.18 (2.08)^a^	83.99 (3.53)^a^	84.04 (3.00)^a^
Ours (Full)	78.01 (11.87)^a^	90.31 (2.20)^a^	84.25 (3.82)^a^	84.19 (3.35)^a^
**Ours (Mid)**	**78.94 (11.88)**	**90.68 (2.16)**	**84.82 (3.34)**	**84.81 (3.17)**

Metrics are shown as mean (standard deviation). mean: averaged Dice scores of all organs/subregions. Bold numbers are the best results

^a^p-value < 0.05 compared with Mid network by paired t-test

**Table 10:** Quantitative results (HD95) of proposed methods compared to state-of-the-art methods for BraTS 2020

**Method**	**HD95**$\downarrow$			
	ET	WT	TC	mean
U-Net [3]	41.35 (113.26)^a^	13.85 (11.23)^a^	18.57 (27.15)^a^	24.59 (45.79)^a^
nnU-Net [33]	35.10 (33.85)^a^	4.89 (2.74)^a^	5.91 (4.86)^a^	15.30 (8.01)^a^
UNETR [26]	26.58 (32.38)^a^	4.18 (2.82)^a^	5.07 (4.05)	12.03 (7.85)^a^
Ours (Base)	29.14 (28.66)^a^	4.77 (2.53)^a^	6.01 (4.12)^a^	13.31 (6.94)^a^
Ours (Full)	26.27 (25.90)	4.56 (2.50)^a^	5.87 (4.32)^a^	12.23 (6.38)^a^
**Ours (Mid)**	**25.22 (25.91)**	**3.65 (2.09)**	**5.02 (3.75)**	**11.30 (6.84)**

Metrics are shown as mean (standard deviation). mean: averaged Dice scores of all organs/subregions. Bold numbers are the best results

^a^p-value < 0.05 compared with Mid network by paired t-test

**Fig. 3:**
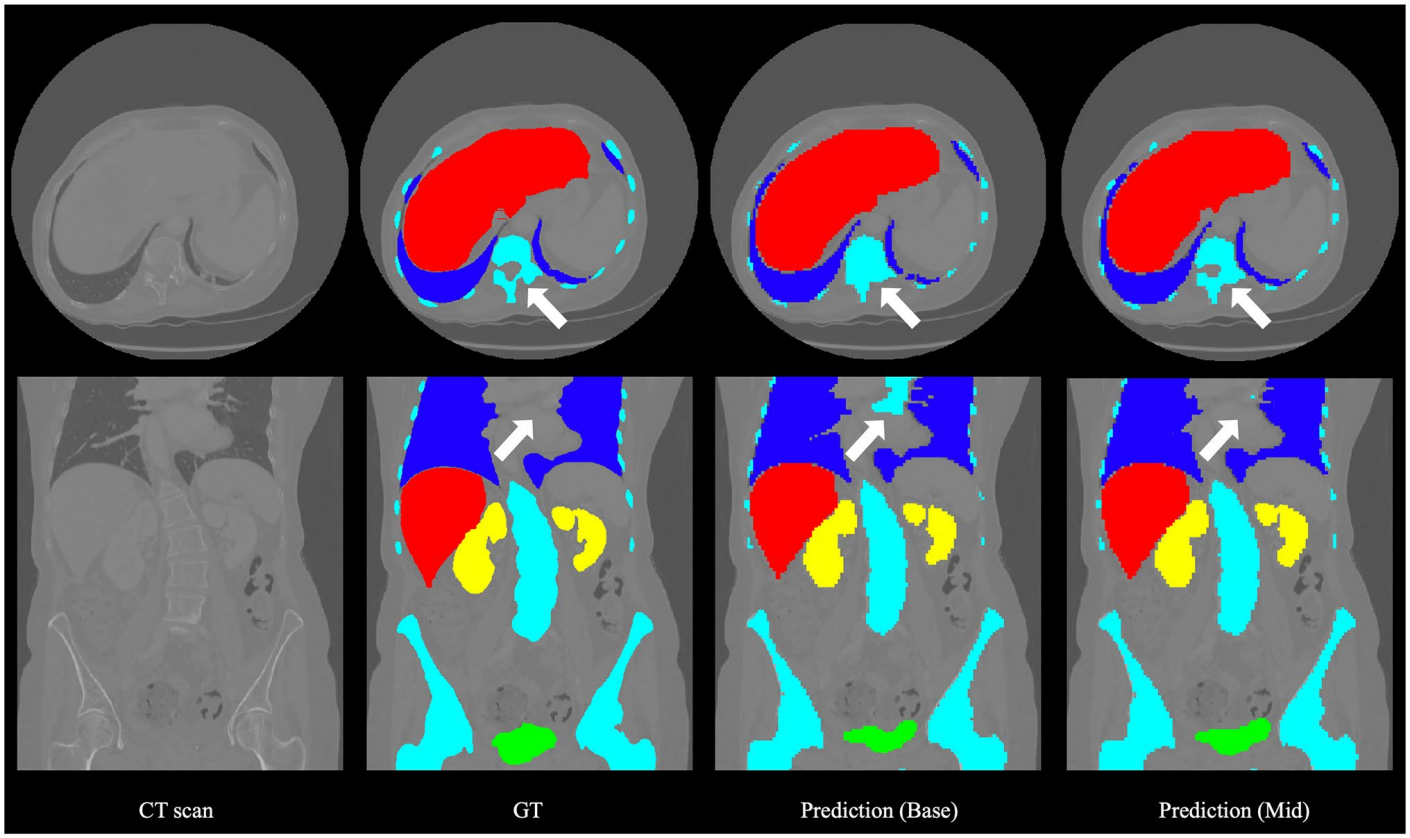
Representative visual results of proposed methods for CT-ORG. From left to right: CT scan, ground truth (GT), and predictions. The labels are liver (red), gladder (green), lungs (blue), kidneys (yellow), and bone (cyan)

**Fig. 4:**
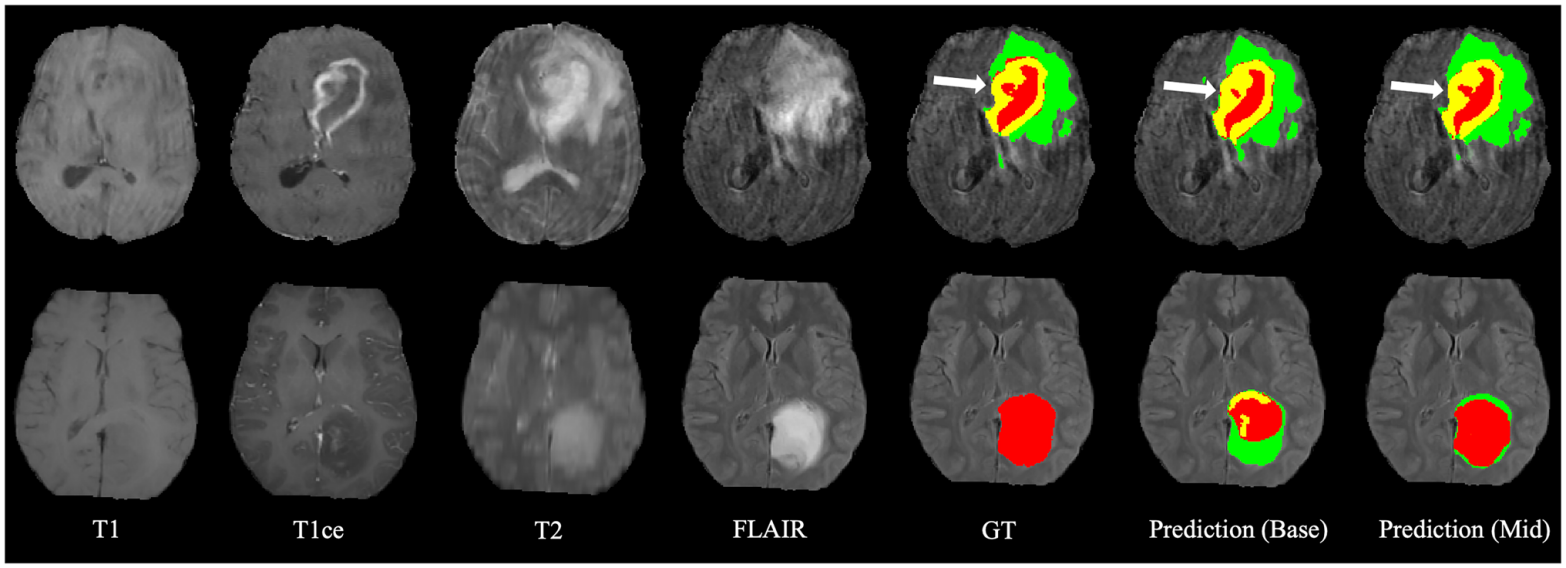
Representative visual results of proposed methods for BraTS 2020. From left to right: four MRI modalities, ground truth (GT), and predictions. The labels are enhancing tumor (yellow), edema (green), and necrotic and non-enhancing tumor (red)

Quantitative results showed that the proposed networks outperformed all state-of-the-art methods in segmenting all organs and tumor subregions, including advanced U-Net (nnU-Net [33]) and Transformer (UNETR [26]). Specifically, the Mid-type network among them was the best-performing approach among them. For multi-organ segmentation, the proposed method achieved the highest Dice score and the lowest HD95 score in all organs, especially the lungs. This might be attributed to the fact that the 3D LK attention module emphasizes lung-related features both spatially and individually, thereby alleviating the problem of overlapping boundaries with other organs, such as the liver. In terms of the Dice score, the Mid network was only slightly inferior to the Base network in segmenting the bladder. We found that adding any attention mechanism would cause an insignificant decrease in Dice for bladder segmentation. This might be due to the uneven distribution of attention to fine organs, resulting in a greater concentration of computing power on others. As for the brain tumor segmentation, the Mid network performed remarkably well regarding ET’s HD95 score, which might also be due to the LK attention module adding feature weights to the correct tumor subregions. Representative segmentation results were also compared visually in Figs. 3 and 4, which further proved the effectiveness of the LK attention module.

**Fig. 5:**
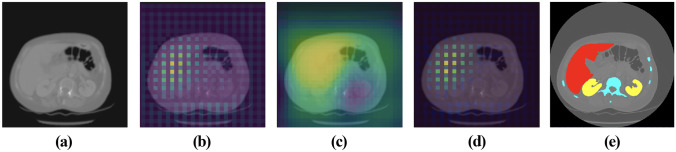
A representative visual effect of the LK attention module. **a** The CT scan input. **b** The upsampled feature map at the middle scale of the decoder. **c** The attention map. **d** The feature map after multiplying with the attention map. **e** The GT labels

Comparing the visual segmentation results of the Base and Mid networks, the performance improvement due to the presence of the LK attention module can be seen (as indicated by pointers). Bones, lungs, ET, and TC had more significant improvements which were also shown in Tables 11 and 12. The improvements brought by the LK attention module on all segmentation targets were statistically validated, except for bladder and ET. The LK attention module might cause an insignificant accuracy decrease in segmenting bladders according to the test. As for the ET, since BraTS 2020 set a penalty of Dice = 0 and HD95 = 373.13 for false positives of ET, the paired t-test cannot verify the change in ET. But overall, this statistic validated the effectiveness of the adaptive feature selection of the LK attention module, as visualized in Fig. 5. In addition, according to Table 6, the performance improvement brought by LK attention only sacrificed negligible efficiency, explicitly increasing the model parameters by only 0.0759%.

**Table 11:** Improvement in quantitative results due to the LK attention module for CT-ORG

	**Dice**$\uparrow$						**HD95**$\downarrow$					
	liver	bladder	lungs	kidneys	bone	mean	liver	bladder	lungs	kidneys	bone	mean
Ours (Base)	95.81	86.81	94.23	92.11	88.20	91.43	1.64	2.83	10.38	2.80	4.93	4.52
Ours (Mid)	96.12	86.48	97.40	92.26	88.51	92.15	1.53	2.93	6.54	2.80	4.12	3.64
Improvement	0.3%	–0.4%	3.4%	0.2%	0.4%	0.8%	–7.1%	3.5%	–37.0%	0.0%	–16.6%	–19.5%
p-value	0.020	0.081	0.040	0.026	0.031	0.030	0.021	0.075	0.025	0.039	0.015	0.028

**Table 12:** Improvement in quantitative results due to the LK attention module for BraTS 2020

	**Dice**$\uparrow$				**HD95**$\downarrow$			
	ET	WT	TC	mean	ET	WT	TC	mean
Ours (Base)	77.94	90.18	83.99	84.04	29.14	4.77	6.01	13.31
Ours (Mid)	78.94	90.68	84.82	84.81	25.22	3.65	5.02	11.30
Improvement	1.3%	0.5%	1.0%	0.9%	–13.4%	–23.4%	16.5%	–15.1%
p-value	0.286	0.013	0.013	0.015	0.095	0.037	0.044	0.65

Furthermore, high-performance deep learning models usually produce incomprehensible results for humans. While these models can yield better efficiencies than humans, it is not easy to express intuitive explanations to justify their findings or to derive additional clinical insights from these computational “black boxes” [52]. Given the importance of explainability in the clinical domain, our proposed LK attention module proved that deep learning models could identify appropriate regions in medical images without overemphasizing unimportant findings. The local explanation furnished directly by the LK attention map (in Fig. 5) argued that there was medical reasoning for the focused parts of the CT scan, which could facilitate clinicians’ decision-making.

### Limitations

Our method still has some limitations. First, as shown in Fig. 3, the segmentation results showed unsmooth edges because their resolution is lower than GT due to resampling. In future work, the resolution of the segmentation mask can be improved by resampling the image to a higher resolution and performing segmentation with sliding windows. Moreover, in the second example of Fig. 4, the TC was not accurately segmented, which might be due to the blurring of the T2 modality. This demonstrates the importance of data integrity for the accurate segmentation of medical images. This can be solved by more diverse data acquisition and data augmentation or by training generative networks to synthesize clear images.

## Conclusion

This paper introduced LK attention for 3D medical image segmentation, which can be easily incorporated into any CNN such as U-Net. The 3D LK attention module combines the advantages of biologically inspired self-attention and convolution, exploits local contextual information, long-range dependencies, spatial and channel adaptation, and uses convolutional decomposition to eliminate the disadvantage of high computational cost. Ablation experiments on the CT-ORG dataset first verified the feasibility of the decomposition of 3D LK convolutions and secondly explored the most efficient deployment design of the 3D LK attention module. The quantitative results of ablation learning indicated that incorporating the 3D LK attention module in the middle of the decoder achieved optimal performance. The Mid-type LK attention-based U-Net achieved state-of-the-art performance on both multi-organ and tumor segmentation compared to advanced CNN and Transformer-based methods. Segmentation results of CT-ORG and BraTS 2020 datasets showed that the 3D LK attention module improved predictions for all organs and tumor subregions except the bladder, especially for lung, ET, and TC. In addition, the 3D LK attention module was proven to be effective in adaptively selecting important features and suppressing noise, which provided local explanations of the model’s prediction. Overall, our method shows promise that can be extended into research in other brain diseases, e.g., ageing and neurodegenerative disorders [53], by combining with transfer learning [54] and graph neural networks [55].

However, some challenges remained. First, the addition of attention caused the scattered computing power for some fine targets such as the bladder. Thus, the LK attention module can be further customized for multi-target segmentation. Second, for large medical images, better sampling or training strategies can be used to further improve the resolution of the segmentation results. Furthermore, since the low quality of the images can significantly reduce the segmentation accuracy, more comprehensive data augmentation and data harmonization [56] strategies and larger training datasets can be considered, or a generative network can be used to synthesize high-quality images [57, 58]. In addition, we plan to validate whether the superior performance of our method also holds in newly released datasets.

## Data Availability

The datasets generated during and/or analyzed during the current study are available from the corresponding author on reasonable request.

## Footnotes

1 CBICA’s Image Processing Portal (https://ipp.cbica.upenn.edu)
